# Using deep transfer learning and satellite imagery to estimate urban air quality in data-poor regions ✩

**DOI:** 10.1016/j.envpol.2023.122914

**Published:** 2023-11-22

**Authors:** Nishant Yadav, Meytar Sorek-Hamer, Michael Von Pohle, Ata Akbari Asanjan, Adwait Sahasrabhojanee, Esra Suel, Raphael E Arku, Violet Lingenfelter, Michael Brauer, Majid Ezzati, Nikunj Oza, Auroop R. Ganguly

**Affiliations:** a Sustainability and Data Sciences Laboratory, Northeastern University, Boston, USA; b University Space Research Association (USRA), Mountain View, USA; c NASA Ames Research Center, Moffett Field, USA; d Imperial College London, London, UK; e University of Massachusetts, Amherst, USA; f University of British Columbia, Vancouver, Canada; g Pacific Northwest National Laboratory (PNNL), Richland, USA; h The Institute for Experiential AI, Northeastern University, Boston, USA

**Keywords:** Deep learning, Air quality, Satellite imagery, Transfer learning

## Abstract

Urban air pollution is a critical public health challenge in low-and-middle-income countries (LMICs). At the same time, LMICs tend to be data-poor, lacking adequate infrastructure to monitor air quality (AQ). As LMICs undergo rapid urbanization, the socio-economic burden of poor AQ will be immense. Here we present a globally scalable two-step deep learning (DL) based approach for AQ estimation in LMIC cities that mitigates the need for extensive AQ infrastructure on the ground. We train a DL model that can map satellite imagery to AQ in high-income countries (HICs) with sufficient ground data, and then *adapt* the model to learn meaningful AQ estimates in LMIC cities using transfer learning. The trained model can explain up to 54% of the variation in the AQ distribution of the target LMIC city without the need for target labels. The approach is demonstrated for Accra in Ghana, Africa, with AQ patterns learned and adapted from two HIC cities, specifically Los Angeles and New York.

## 1 Introduction

**Fig. 1:**
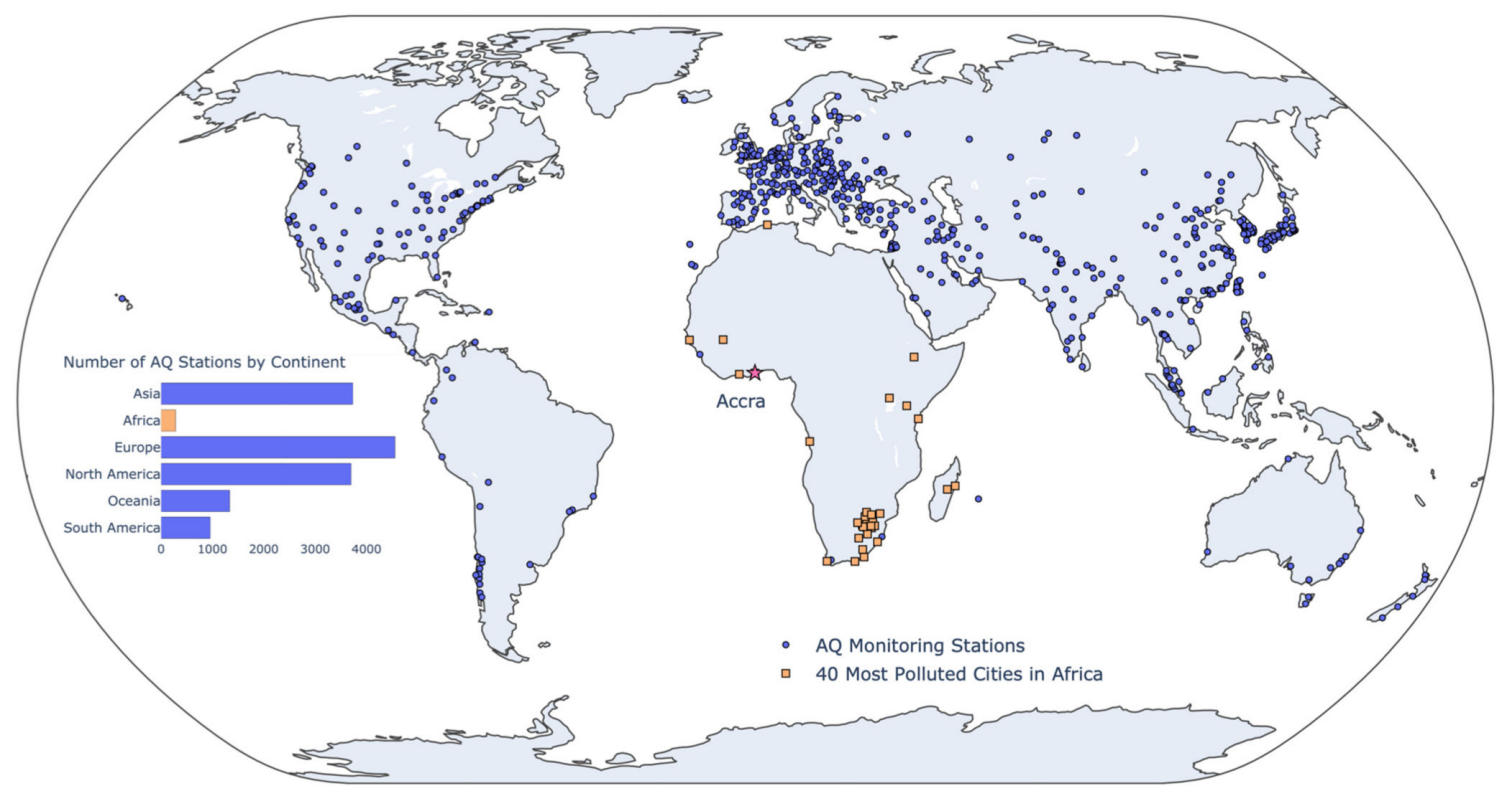
Skewed distribution of air quality (AQ) monitoring stations across the globe. The figure shows 681 cities reporting AQ data to the World Air Quality Index (WAQI) Project through public, private, and citizen efforts. Out of approximately 15,000 stations, less than 300 are located in Africa. The third most polluted city in Africa - Accra, Ghana - has only one monitoring station contributing to WAQI compared to 20+ stations for New York City, as an example. (For interpretation of the colors in the figure(s), the reader is referred to the web version of this article.)

Accurately estimating urban air quality (AQ) is vital for effective policy and research. However, not all parts of the world are equally equipped with an adequate ground monitoring network (see Fig. 1). Cities in low- and middle-income countries (LMICs), such as in sub-Saharan Africa and South-East Asia, severely lack the infrastructure required to monitor AQ (Martin et al., 2019) at policy-relevant scales. Of the approximately 15,000 AQ stations reporting to the World Air Quality Index (WAQI) Project, less than 300 stations are located in Africa. Only 7 out of 54 countries in Africa have real-time AQ monitoring stations (UNICEF, 2019) - with many major cities having one or fewer stations. For example, in Greater Accra Metropolitan Area (GAMA), Ghana (the city of interest in this work), the only publically available AQ monitoring site is located at the US Embassy in Ghana. In comparison, in the Greater New York Area (GNYA) - one-fourth the size of GAMA - more than 30 monitoring sites report to the US Environmental Protection Agency (EPA). As urbanization increases, the economic burden of urban air pollution will be immense (Fisher et al., 2021; Roy, 2016). According to a 2016 OECD report (OECD, 2016), the estimated economic loss due to air pollution is $2.6 trillion annually. LMICs are expected to be disproportionately affected given the infrastructure constraints and presence of more polluted and densely populated urban areas (Amegah and Agyei-Mensah, 2017).

Our inability to estimate AQ accurately in LMIC cities hinders future preparedness and risk mitigation (Coker and Kizito, 2018). Traditional methods for AQ monitoring are resource-intensive and expensive to scale. Where physical models are too coarse (> 4 km resolution) (Appel et al., 2013), one of the most widely used methods for characterizing AQ distribution at urban policy scales is land-use-regression (LUR) modeling. (Lee et al., 2017; Jin et al., 2019). LUR models solve multiple regression equations that map sample locations and different environmental variables. It requires gathering locally measured data on traffic, weather, land use, and population density. The resulting models can predict AQ levels at unmeasured locations with a high spatial resolution (∼ 100 m). However, data collection for LUR models is time-consuming and may even be cost-prohibitive if requisite data is unavailable. Thus, LUR models are available for only a handful of urban regions, mainly in high-income countries (HICs) (Johnson et al., 2010). In contrast, remote sensing can be a viable alternative that can be harnessed for urban AQ estimation. Satellite imagery, at increasingly high resolution (HR), is globally available from both government and commercial resources. Simultaneously, the advances in data-driven methods such as machine learning (ML) - particularly deep learning (DL) - provide a promising approach to generating non-trivial insights from satellite imagery. As a result, recent works have combined ML / DL techniques with satellite imagery for regions where traditional data sources are limited or unavailable. (Zhu et al., 2017; Yuan et al., 2020; Rolf et al., 2021; Oshri et al., 2018; Yeh et al., 2020; Yadav, 2022).

Here we present a new DL-based method (DeepAQ hereafter) for learning AQ spatial distribution (in terms of annual mean *NO*_2_ levels in μg/m^3^) over data-poor regions using high-resolution satellite imagery (HRSI). We put forward and evaluate two hypotheses: first, HRSI is indicative of AQ information, and a DL model can be trained to map satellite imagery to AQ estimates over a region. The second hypothesis - which is the ultimate goal of this work - is that such trained ML/DL models can be applied/transferred to new test cities in data-poor LMICs. We choose *NO*_2_ as the pollutant of interest in this study since we are interested in capturing the intra-city variability at meter-scale. Our approach is equally applicable to other air pollutants; however, for certain pollutants such as ***PM***_2.5_, the intra-city variability may not be high enough.

Although there are existing methods using satellite-derived products for AQ estimation (e.g., mapping OMI-*NO*_2_ vertical column to ground-level *NO*_2_, (Kang et al., 2021)), they are too coarse (> 3 km) to capture the high local spatial variability of ground-level *NO*_2_ at meter-scale. The proposed technique takes a fundamentally different approach to solving AQ estimation at meter-scale. It directly maps visual satellite imagery to AQ estimates on the ground using a computer vision-based (CV) technique. Using CV, we can detect visual features in a satellite image that can inform us of the AQ over that region. To provide a simplistic example, a satellite image containing a dense road network may indicate a high concentration of air pollutants such as *NO*_2_; an image comprising a green cover may suggest the opposite. After the model extracts meaningful features from an image patch, it then maps it to the appropriate AQ level based on the set of features learned. This learning - including feature extraction and estimation - happens end-to-end during training in DeepAQ.

The main advantage of the proposed approach is global coverage with a focus on data-poor LMICs and AQ estimates at very high spatial resolutions (∼ 200 m). Furthermore, once the model is trained, subsequent inferences are expected to be inexpensive.

## 2 Methodology and data

Before we apply DL-based models for AQ estimation in data-poor LMIC cities, we must overcome a circular challenge. DL models tend to perform well in supervised settings where large labeled training data is available, but generalization to new domains (datasets) remains poor (Neyshabur et al., 2017; Wilson and Izmailov, 2020). Because LMICs lack training data in the first place, this limits the direct application of ML / DL techniques to satellite imagery over regions - usually high-income and developed - with abundant ground-level data for model training and validation. We propose to overcome this challenge using a transfer learning approach (Zhuang et al., 2020; Tan et al., 2018). In ML, transfer learning is the idea that knowledge gained while solving one problem can be applied to a different but related problem.

### 2.1 Transfer learning

We define transfer learning (TL) using the framework in Pan and Yang (2010). A domain 𝒟 = {χ, 𝒫 (χ} consists of a feature space *χ* and a marginal probability distribution 𝒫(χ). Given a domain, a task 𝒯 = {𝒴, *f*(⋅)} consists of a label space 𝒴 and a predictive function *f*(·) which models 𝒫(y|x) for y ∈ 𝒴 and *x* ∈ *χ*. Given a source domain 𝒟*_S_* and learning task 𝒯*_S_*, and a target domain 𝒟_𝒯_ and learning task 𝒯_𝒯_, transfer learning aims to improve the learning of the target predictive function *f_T_*(·) in 𝒯_𝒯_ using the knowledge from 𝒟*_S_* and 𝒯*_S_*. In this work, the source and target domains are the cities in HICs and LMICs, respectively; and the target task is to estimate AQ in the target domain (LMICs).

#### 2.1.1 Unsupervised transfer learning or ‘domain adaptation’

Transfer learning is an umbrella term - based on whether 𝒟*_S_* ≠ 𝒟_𝒯_, 𝒯*_S_* ≠ 𝒯_𝒯_ or both, it can be categorized into different sub-types. A common scenario arises from the phenomenon known as dataset shift or *domain shift* (Bu et al., 2020), where the marginal probability distribution of the feature space *χ* in source domain 𝒫*_S_*(χ) is different from the target domain, 𝒫_𝒯_(χ). As a result, the models trained on the feature representations from one domain do not generalize well to new datasets (domains). The typical solution is to use the model trained on one domain and fine-tune it using limited labeled data from the target domain. However, it might be prohibitively difficult to obtain enough - if any - labeled data from the target domain to properly fine-tune the large number of parameters employed by DL models. Such a problem of adapting an existing model to a new dataset (domain) in the absence of target labels is called Unsupervised Domain Adaptation (UDA) (Baktashmotlagh et al., 2013), which is also the focus of this work. A comprehensive overview of transfer learning approaches is presented in Pan and Yang (2010).

UDA is essentially a representation learning problem across domains. In DL, feature representation learning is a set of techniques that allows a DL model to learn the hidden representations (features) from raw data needed to solve a downstream task such as classification. In UDA, the goal is to learn a joint feature space that is domain-invariant. In other words, the UDA model maps the source and target features into a common feature space to ensure that the model cannot distinguish between the training and test domain data samples. By doing so, the model is expected to perform better on feature embeddings and, ultimately, the downstream task of the target domain. For more theoretical details on UDA, readers are directed to Wilson and Cook (2020).

### 2.2 Proposed approach: DeepAQ

In this work, we propose an unsupervised transfer learning approach called DeepAQ and demonstrate it for the city of Accra in Ghana, Africa. The DeepAQ model performs two steps: first, a DL model is trained to estimate annual mean *NO*_2_ levels at 200 m resolution over cities with sufficient training data. Los Angeles and New York City (NYC) are chosen as two candidate cities because of 1) the availability of labeled data and 2) a wide distribution of *NO*_2_ levels and associated patterns. In the second step, the DeepAQ model is transferred to Accra in a completely unsupervised setting, i.e. labeled data from Accra is not required during model training. For performance validation, we use the AQ data collected by our team across 10 fixed sites in Accra for one year (Clark et al., 2020). The sites were selected to represent a range of land uses and sources such as road traffic, commercial, industrial, and residential areas, and various neighborhoods with diverse socioeconomic classes. We must add that even though we mention two steps, the entire DeepAQ model training and transfer learning happens in a single end-to-end regime.

#### 2.2.1 Model architecture and training

**Fig. 2:**
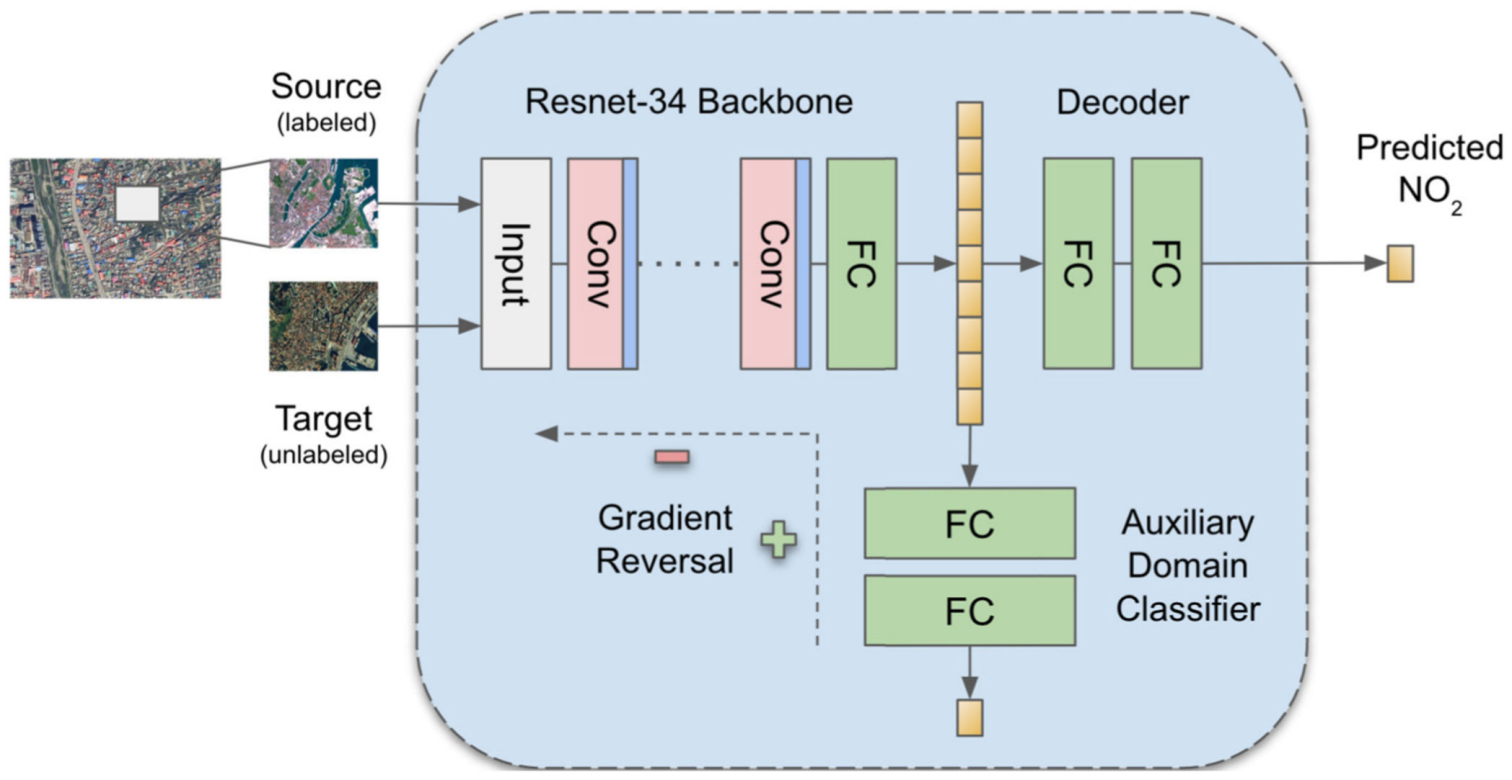
Schematic Architecture for DeepAQ. The three main components include a ResNet-34 feature encoder, an auxiliary domain classifier, and a fully-connected decoder.

The DeepAQ model is a CNN-based Generative Adversarial (GAN) Model (Fig. 2) (Ganin and Lempitsky, 2015). The DeepAQ architecture comprises three components - a ResNet-34 (He et al., 2016) feature encoder (G), a 3-layer fully-connected decoder (D), and a 3-layer fully-connected auxiliary domain classifier (critic, C). During the training stage, the encoder takes two images as input, one each from the source and target domains, and generates corresponding feature embeddings. In the next step, critic C takes the feature embeddings generated by G and attempts to classify them as coming from the source or target domain. At the same time, the decoder (D) takes the feature embeddings to estimate the target value. The overall loss function includes two losses: first, a regression loss that optimizes D and G to decrease the error in estimating the target value; second, an adversarial loss (Tzeng et al., 2017) that trains C and G together, where the goal of G is to generate embeddings that can fool the domain critic C. In other words, the adversarial loss forces G to generate domain-invariant feature embeddings that the critic C cannot classify correctly. Achieving domain invariance is critical for successfully transferring the model to the target domain (city) (Ganin and Lempitsky, 2015). The entire training (for both the losses) progresses simultaneously for a certain number of iterations till the critic C is completely fooled, i.e., its performance in classifying the feature embeddings is no better than a random choice. At inference, critic C is detached from the network; the model takes an input image from the target domain, which passes through G and D to generate the final output - the mean annual *NO*_2_ corresponding to that image patch.

### 2.3 Design of experiments

We examine two connected hypotheses: first, a DL model can be trained to estimate the AQ distribution over a region using HRSI (no transfer learning yet). Second, we argue that such a model can be applied (transferred) to new regions of interest using unsupervised transfer learning. To validate the first hypothesis, we train a ResNet-34 model (He et al., 2016) using training and test data from LA. ResNet-34 is a commonly used CNN. The architecture includes multiple convolutional layers (called the backbone) stacked together and a fully-connected layer at the end (called the head or decoder) to generate the desired output. The backbone takes an image patch as input and generates a feature embedding, which then passes through the head resulting in the final output. In our case, the model takes a 200 * 200*m*^2^ image patch as input and outputs a point value, the mean annual *NO*_2_ over that patch.

For the second hypothesis, we conduct two experiments. Since we have minimal labeled data points in Accra, we cannot directly validate the efficacy of DeepAQ in Accra. Therefore, as an initial *proof-of-concept*, we demonstrate the performance of DeepAQ by transferring the model from LA (source) to NYC (target), where we have adequate labeled data for validation. The labeled data from NYC is only used for validation and not for training DeepAQ. During training, the DeepAQ model takes as input two image patches - one from the source city and another from the target city. At inference, the trained model predicts the *NO*_2_ levels over the target city (NYC). In the second experiment, we use both LA and NYC as source cities and transfer the DeepAQ model to Accra, Ghana.

In summary, we conduct a total of three experiments (Table 1). In each experiment, the baseline model is a ResNet-34 CNN trained only on the source city (or cities) data with no exposure to images from the target city. Two metrics are used to evaluate performances - normalized root mean squared error (*nrmse*) and coefficient of determination (*R*^2^).

### 2.4 Data

#### Satellite Imagery

Over 300 raw satellite images from MAXAR World-View2 (WV2) are used in this study to train and validate the DeepAQ model. WV2 was launched in 2009 as part of the MAXAR satellite constellation. It is a sun-synchronous satellite located 770 km from Earth. Images are produced in eight spectral bands in the VIS–NIR range (400 nm−2500 nm) with a spatial resolution of 2m (rescaled to ∼ 2.5 m to better match the target data resolution). A single raw image roughly spans an area of 400 km^2^. In this work, only the visible (RGB) bands are used. From each image, multiple 80 × 80 pixel patches are extracted, such that each patch covers a 200 m × 200 m region to match the target data resolution. In total, approx. 40, 000, 16, 000, and 28, 000 image patches are generated for LA, NYC, and Accra, respectively.

#### Target Data

The mean annual *NO*_2_ target data (μg/*m*^3^) at 200 m resolution is available from (2010) LUR models for LA and NYC (Bechle et al., 2015). LUR models solve multiple regression equations that map sample locations and various environmental variables. It requires gathering locally measured data on traffic, weather, land use, and population density, among others. LUR data is only available for a select few cities as LUR models require substantial time and effort. They commonly serve as inputs for environmental policy and research, as it is not possible to obtain station data at such high resolution. For Accra, a team of researchers associated with this work collected *NO*_2_ ground-level data across 140 different locations for a year (Clark et al., 2020). Although in DeepAQ, no labeled data from Accra is used, the 140 data points collected serve to validate the predictions over Accra.

#### Auxiliary Input for Local-Attention

The DeepAQ model performs two tasks: extracting meaningful features from an image patch and mapping it to the appropriate AQ level based on the set of features learned. This learning - including feature extraction and regression - happens end-to-end during the model training. As the satellite image patches are fed into the model as i.i.d. samples, the model cannot differentiate between similar-looking patches but with different *NO*_2_ labels. For instance, the DeepAQ model will equate all patches containing similar green covers with roughly equal AQ levels, which may not be the case in real life. A small green cover in the downtown region might have a different AQ level than one in the suburban area. Hence, we need to provide additional context to the model. The distance of each patch from the nearest major road is provided as an auxiliary input to DeepAQ to induce localized attention during model learning. Since proximity to roads strongly correlates with *NO*_2_ levels, the model will attend to nearby patches, with similar values for the auxiliary input, for predicting the *NO*_2_ value over the input patch in question. The road network information for the three cities is obtained from OpenStreetMap (OpenStreetMap, 2017), a provider of freely available global geographic databases.

#### Data Pre-Processing

Raw satellite data need to be processed before use. In this work, radiometric correction, followed by filtering for cloud cover, was performed on the satellite images. Finally, the images were normalized between the range of (−1, 1) before patch extraction.

## 3 Results

**Fig. 3:**
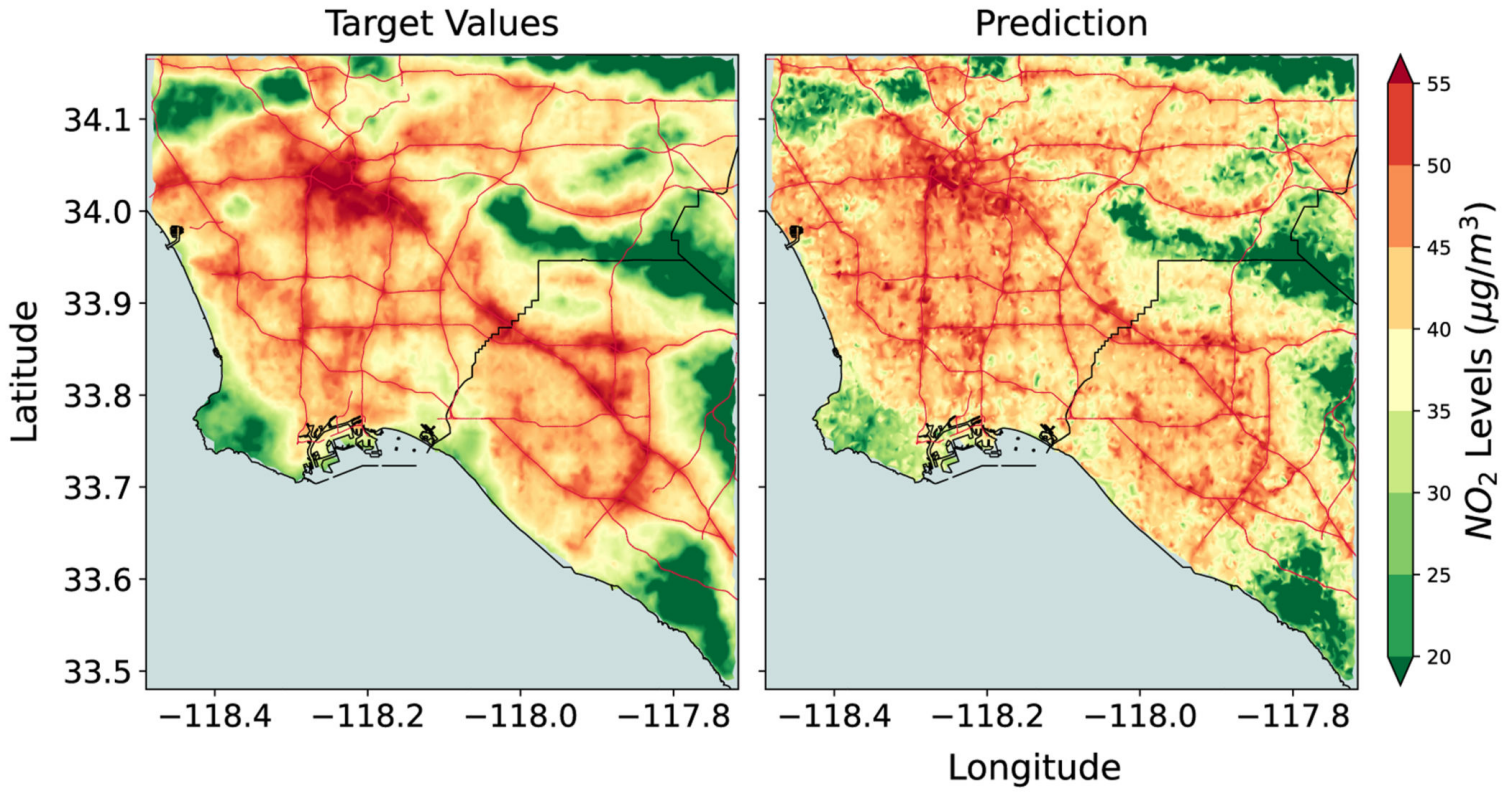
Mean annual *NO*_2_ levels (μg/m^3^) over the Los Angeles Area (LA) as estimated by the ResNet-34 model. The model is trained and tested using data from LA only. The model is able to identify regions of low and high *NO*_2_ levels with high accuracy (also see Supplementary Fig. S3). It validates the primary hypothesis of this work, i.e., a deep learning-based method for AQ estimation can be developed. The road network is superimposed on top (red lines) for reference.

**Fig. 4:**
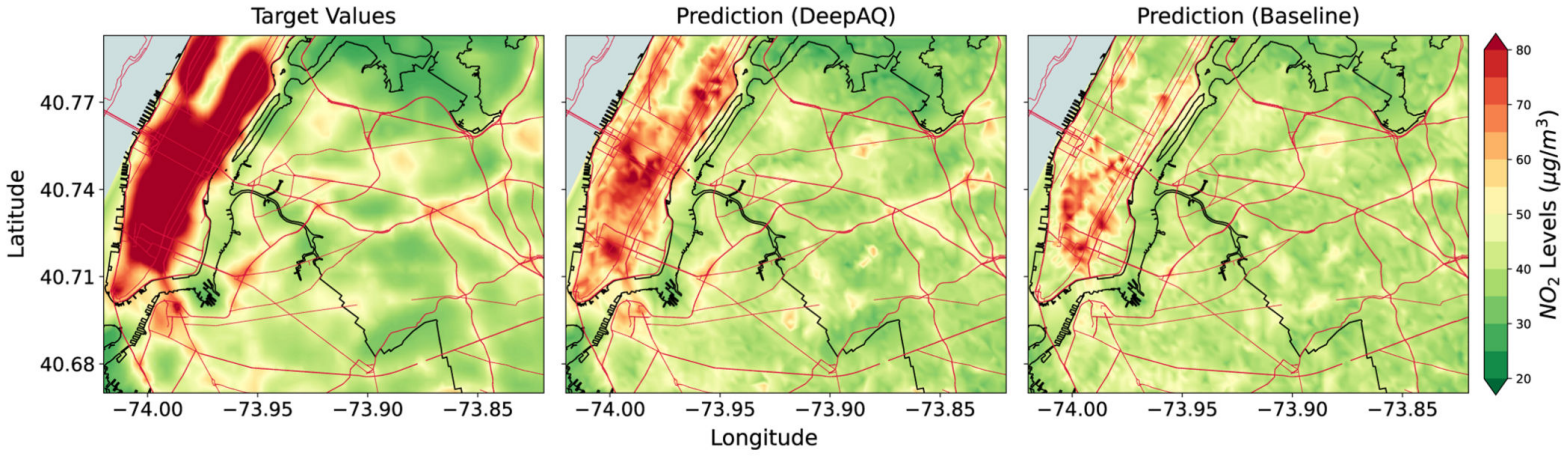
Mean annual *NO*_2_ levels (μg/m^3^) over New York City (NYC) as estimated by DeepAQ, and compared with the baseline ResNet-34 model (trained only on Los Angeles (LA) data with no transfer learning). In this case, LA acts as the source city (domain). Compared to the baseline, the DeepAQ model is able to better identify regions of high *NO*_2_ levels, such as in the Manhattan area of NYC (upper left). The road network is superimposed on top (red lines) for reference. See Supplementary Fig. S3 for the case when no road network information is provided to the model.

**Fig. 5:**
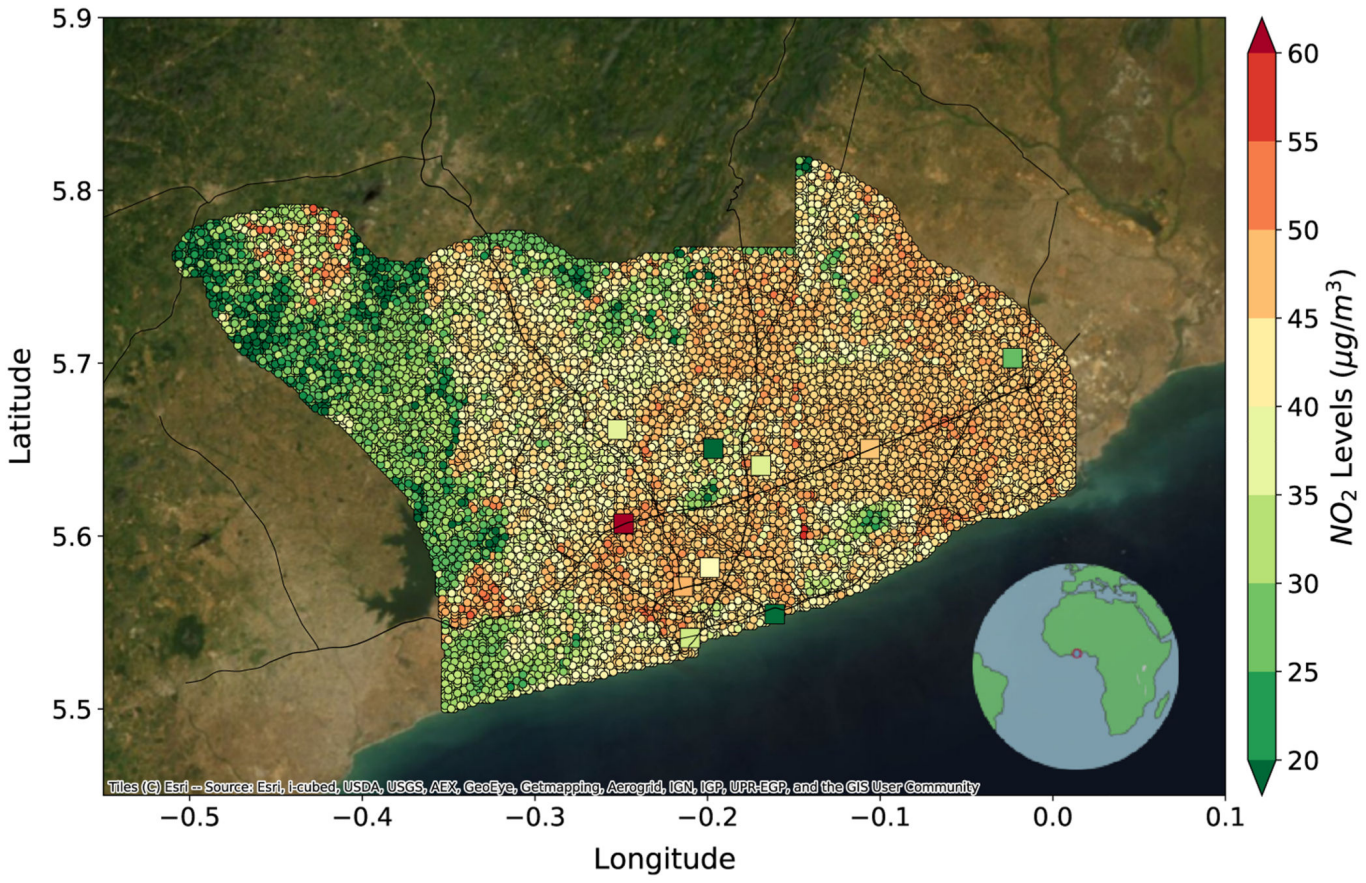
Mean annual *NO*_2_ levels (μg/m^3^) over Accra, Ghana, as predicted by DeepAQ. The square dots point to the fixed monitoring stations measuring *NO*_2_ levels across Accra. The DeepAQ model is able to capture the spatial distribution of *NO*_2_ over Accra, with higher levels observed in the city center and near the highways. See Supplementary Fig. S4 for the case when no road network information is provided to the model.

The main results corresponding to the three experiments conducted in this study are presented in Figures 3–5. In the first experiment, we train a ResNet-34 Convolutional Neural Network (CNN) (He et al., 2015) using data from LA only. We observe the trained model can estimate the spatial distribution of annual *NO*_2_ with high accuracy (Fig. 3) - achieving an *R*^2^ score of 0.806 (Fig. S1) and a correlation coefficient (*r*) of 0.91. The *R*^2^ score represents the variance in the true values explained by the predicted value. A high *R*^2^ indicates that the CNN model can successfully retrieve the spatial pattern in the dataset. In other words, there is a meaningful signal in the dataset (in terms of visual features) that the ResNet-34 model is able to capture and map to appropriate *NO*_2_ estimates. For instance, in Fig. 3, we find the model can predict high *NO*_2_ values in the downtown region (top left). In section 3.1 (DeepAQ Model Interpretation), we will also understand what the model learns from the satellite imagery to arrive at its predictions. Based on the results, we state that a DL model can indeed be trained to map satellite imagery to AQ over a region (hypothesis 1; see Table 1).

For the second and third experiments, we use the ResNet-34 model (trained only on source city (cities)) as a baseline model to compare the performance of DeepAQ. The baseline model provides us with the lower bound of the model performance, i.e., the DeepAQ model should perform at least as well as the baseline (when no knowledge transfer occurs). In the *proof-of-concept* experiment (LA → NYC), we observe the DeepAQ model substantially outperforms the baseline (Fig. 4). It reduces the *nrmse* by 32%, and the *R*^2^ score is improved by ∼ 2.7 times (Table 2, and Supplementary Fig. S2). Thus, the DeepAQ model can explain 2.7 times more variance in the AQ distribution in NYC than the baseline. We argue that the model can learn semantically meaningful features that can be transferred across cities and are indicative of AQ estimates. In comparison, the baseline performs poorly outside its training domain. Visually, the difference in model performance can be seen in Fig. 4. The DeepAQ model is able to capture the higher *NO*_2_ spatial distribution over the Manhattan region better than the baseline, which tends to converge toward the average value. However, the DeepAQ model struggles to predict areas with very high *NO*_2_ values (> 100*ug*/*m*^3^). The result is consistent with the literature, which finds that ML models struggle with extreme values in long-tailed data distributions. A simple explanation is that few extreme data points are available for the model to learn effectively. In this case, the distribution of *NO*_2_ values rarely goes above 65*ug*/*m*^3^ for the source city (LA), which might explain why the DeepAQ model struggles with extremely high values.

We also provide auxiliary information to the DeepAQ model in the form of the distance of each image patch from the nearest major road. The idea of inputting auxiliary information is motivated to induce localized attention-based (Li et al., 2017) learning in the model. In the absence of the auxiliary input, each satellite image acts as an i.i.d. sample. The model cannot meaningfully differentiate similar-looking patches from locations with different *NO*_2_ values. For instance, an image patch over a park in downtown NYC should differ in *NO*_2_ value from one in the suburbs. Using the auxiliary input, the DeepAQ model attends to nearby image patches with similar road distances while predicting the *NO*_2_ value for a given image patch. For more granular analysis, different weights might be assigned to various types of roads. However, in our experiments, it did not improve the performance much. To tease out the impact of the auxiliary input, we also present results when no road network information is provided to the DeepAQ model and the baseline model (see Supplementary Fig. S3). As expected, the predictive performance deteriorates for both models. While the baseline model completely loses its predictive power, the DeepAQ model can still characterize the spatial distribution of *NO*_2_ over NYC. The DeepAQ model is able to localize acute regions of high *NO*_2_ values, such as road junctions, which the baseline is unable to identify.

In the third experiment, for Accra, we consider both LA and NYC as source cities. The baseline model is trained on the combined data from the source cities and tested on Accra without any transfer learning. Since we only have 10 labeled data points for Accra, we perform a 1000-sample bootstrap analysis and take the mean value of a 3×3 grid closest to the labeled data point for validation. The DeepAQ model reduces the *nrmse* over the baseline by 26% and improves the *R*^2^ by 1.9 times over the baseline. Furthermore, the DeepAQ model can explain roughly 54% of the variance in the AQ distribution over Accra. We observe the model can associate the central Accra metropolis (downtown) region with a high *NO*_2_ concentration (Fig. 5). DeepAQ also identifies the protected green cover regions named Achimota Forest (north central) and Densu Delta Area (southwest) as regions of low *NO*_2_ values. Further, areas adjacent to the highways are marked with higher *NO*_2_ concentrations due to vehicular traffic. The performance of DeepAQ in the absence of auxiliary road network information is presented in Supplementary Fig. S4. The corresponding *R*^2^ plots are presented in Fig. S5.

Based on the quantitative results, we find one of the major limitations of DeepAQ, in its current form, is the underestimation of extremes. The simplest explanation is that there are fewer samples at the extremes for the model to train. On a deeper note, DeepAQ is trained to learn a shared feature space between the source and target cities. If the AQ generation process, and as a result the AQ distribution, is vastly different between the source and target cities, the shared feature space will contain proportionately less predictive information. For example, the AQ distributions of LA and NYC are compared in Fig S6. We suggest two research directions to mitigate this issue: 1) with a diverse set of source cities to train, the model can learn a broader set of shared features, and 2) bias-correction, i.e. if limited labeled data from the target city is available, the model can be fine-tuned in a semi-supervised setting for improved performance. Lastly, heuristic techniques such as using a modified loss function and data augmentation might further enhance the model performance (Shorten and Khoshgoftaar, 2019). In this work, we have purposely considered the strongest constraint that no ground data is available in the target city to focus on validating the fundamental hypotheses proposed earlier.

### 3.1 DeepAQ model interpretation

Deep neural networks (DNN) such as DeepAQ can be good at making predictions, but their decisions are hard to explain in ways that humans can easily understand (Gunning et al., 2019; Jiménez-Luna et al., 2020). Therefore, in addition to evaluating the performance of DeepAQ in terms of metrics, we want to know if the model is able to learn meaningful high-level features that are indicative of *NO*_2_ levels, for example, roads, buildings, and parks. Although it is hard to unravel the exact high-dimensional mapping the DeepAQ model constructs to make a prediction, we can understand the model behavior using pixel attribution-based methods or saliency maps (Adebayo et al., 2018). Saliency refers to unique features (pixels) of the image that help with visual processing. These unique features depict the visually important locations in an image. In the context of model interpretation, saliency maps highlight the pixels in the input image the DNN deems relevant for making a prediction. Here we use a well-known saliency map-based method called Grad-CAM (Selvaraju et al., 2017) to interpret the DeepAQ model output. The Grad-CAM technique utilizes the output gradients with respect to the final convolutional feature map to identify the parts of an input image that most impact the output score. The output gradients vis-a-vis different model layers are calculated during backpropagation. The regions in the convolution map where this gradient is large contribute maximally toward the model output. What the corresponding pixel regions denote in the input image can help us understand how the DNN model extracts information from the input.

**Fig. 6:**
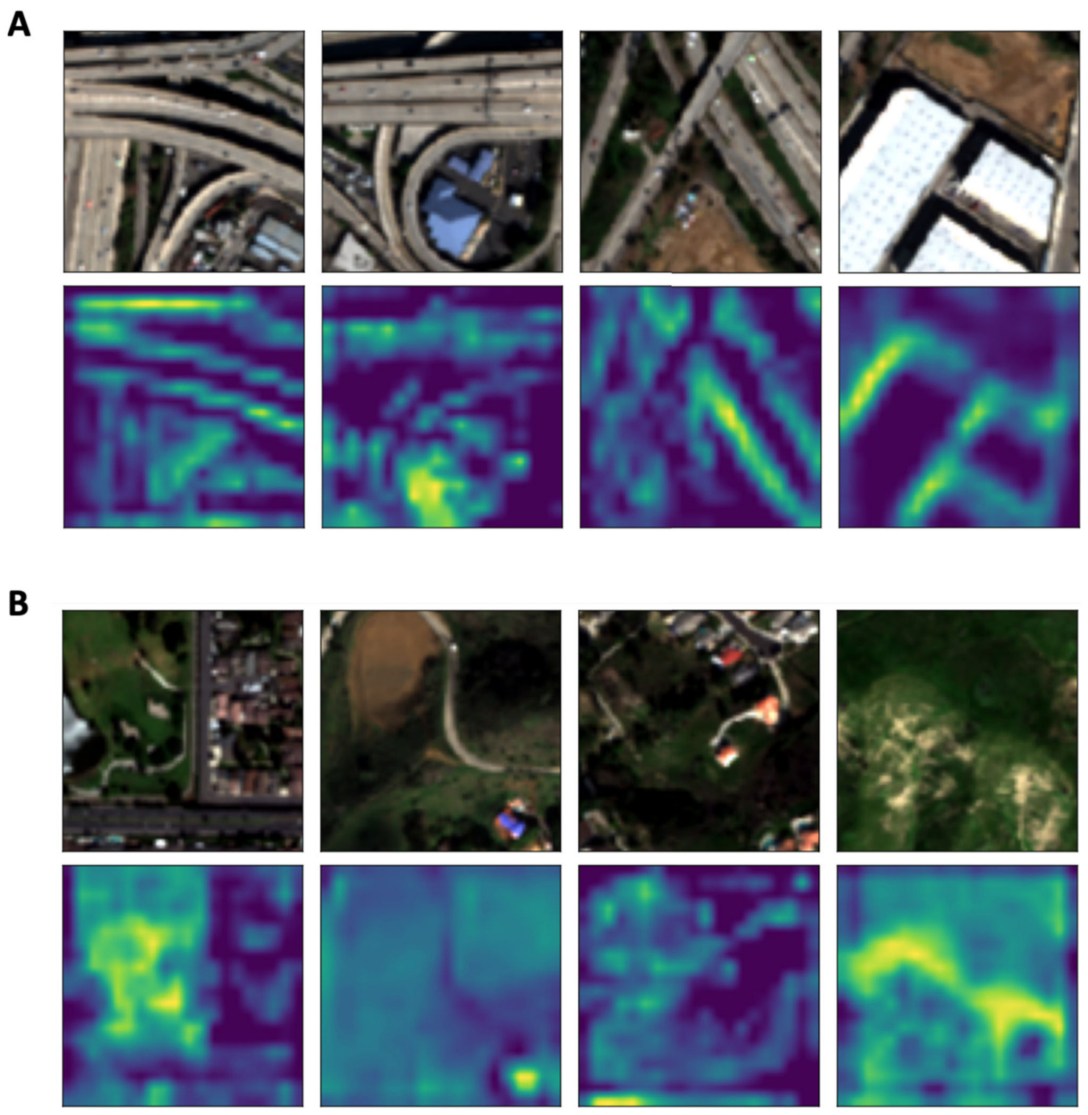
Interpreting the DeepAQ model. Using the Grad-CAM method, regions of the input that are considered important by the model for prediction are visualized. The top row in (A) and (B) contains four satellite image patches from Los Angeles with high and low *NO*_2_ levels (μg/m^3^), respectively. The bottom row contains the pixel-importance maps generated using Grad-CAM. Pixels in yellow refer to the pixels that maximally contributed to the DeepAQ output against that image patch. The DeepAQ model is able to learn meaningful high-level features such as freeways and green cover and relate them to different levels of *NO*_2_.

For our analysis, we select two sets of images corresponding to high and low levels of *NO*_2_ and generate the pixel-level saliency maps using Grad-CAM for each image (Fig. 6). The pixels that light up correspond to regions the DeepAQ models considered relevant for their prediction. For instance, in Fig. 6A, the pixels denoting freeways or roads are highlighted - the DeepAQ model learns this information and predicts high *NO*_2_ values for such regions. Similarly, in Fig. 6B, for areas with low *NO*_2_ values, the DeepAQ model is able to identify them as green covers. We conclude that the DeepAQ performance is due to its ability to learn semantically meaningful and potentially generalizable features that are critical for successful domain adaptation.

## 4 Conclusion

Poor urban air quality (AQ) is an imminent health hazard with dire socio-economic consequences, especially in data-poor low and middle-income countries (LMICs). Traditional methods for high-resolution urban AQ estimation are resource-intensive and expensive to scale. We propose a novel deep transfer learning based approach that uses globally available satellite imagery for AQ estimation in LMIC cities. Results suggest that the proposed method can generate reasonable AQ estimates for LMIC cities at policy-relevant scales. To our knowledge, this is the first work capable of generating continuous AQ maps at an extremely high resolution (∼ 200 *m*) without the need for (labeled) ground data in the region of interest. We also reiterate that this work should not be seen as an alternative to retrieval-based approaches (Handschuh et al., 2022) that operate on km-scale. Instead, it is intended to be complementary to Land-Use-Regression (LUR) models. Our approach is globally scalable and mitigates the need for extensive AQ monitoring infrastructure, making it particularly amenable for developing regions. For the AQ-ML community, DeepAQ is expected to serve as a methodological advance to build upon.

At the same time, an AQ estimation approach based solely on satellite imagery has its limitations. Not all explanatory factors that affect a region’s air quality can be observed in a satellite image, for instance, the local weather. However, such information can be added as an auxiliary input to the model for improved performance, similar to how we used road distance as an additional source of information for the model. A future line of research would be to work with domain experts to combine local information with generalizable pattern-trained deep-learning models. Thus, DeepAQ must be viewed as a means to alleviate and not completely replace the need for ground-based data, as local data can be used to further fine-tune the model performance for individual regions. As satellite imagery with even higher resolution becomes available, the performance of DeepAQ-type models is expected to improve. Lastly, although the focus of this work is AQ estimation, the underlying fundamental approach of DeepAQ can be applied to a variety of challenges where traditional sources of data are unavailable.

## Supplementary Material

Supplementary information

## Figures and Tables

**Table 1:** List of the experiments conducted in this study. Cities marked with ** indicate the target cities as described in the DeepAQ methodology. No labeled data from the target cities are used during training.

Hypothesis	Experiment	Data		Transfer Learning
		Training	Testing	
1	1	LA	LA	No
2	2	LA + NYC**	NYC	Yes
2	3	LA + NYC + Accra**	Accra	Yes

**Table 2:** Results. DeepAQ compares favorably to the baseline model, which is a standard CNN model (ResNet-34) with no transfer learning component.

City	NYC		Accra	
Method	*nrmse*	*R* ^2^	*nrmse*	*R* ^2^
DeepAQ	**0.202**	**0.642**	**0.297**	**0.541**
Baseline	0.293	0.243	0.401	0.276

## Data Availability

The data that support the findings of this study are available from MAXAR, but restrictions apply to the availability of these data, which were used under license for the current study, and so are not publicly available.

## Code availability

The code used to generate the results can be made available upon request.

## References

[R1] Adebayo J, Gilmer J, Muelly M, Goodfellow I, Hardt M, Kim B (2018). Sanity checks for saliency maps. Adv Neural Inf Process Syst.

[R2] Amegah AK, Agyei-Mensah S (2017). Urban air pollution in sub-Saharan Africa: time for action. Environ Pollut.

[R3] Appel K, Pouliot G, Simon H, Sarwar G, Pye H, Napelenok S, Akhtar F, Roselle S (2013). Evaluation of dust and trace metal estimates from the community multiscale air quality (cmaq) model version 5.0. Geosci Model Dev.

[R4] Baktashmotlagh M, Harandi MT, Lovell BC, Salzmann M (2013). Unsupervised domain adaptation by domain invariant projection.

[R5] Bechle MJ, Millet DB, Marshall JD (2015). National spatiotemporal exposure surface for no2: monthly scaling of a satellite-derived land-use regression, 2000–2010. Environ Sci Technol.

[R6] Bu K, He Y, Jing X, Han J (2020). Adversarial transfer learning for deep learning based automatic modulation classification. IEEE Signal Process Lett.

[R7] Clark SN, Alli AS, Brauer M, Ezzati M, Baumgartner J, Toledano MB, Hughes AF, Nimo J, Moses JB, Terkpertey S (2020). High-resolution spatiotemporal measurement of air and environmental noise pollution in Sub-Saharan African cities: pathways to equitable health cities study protocol for Accra, Ghana. BMJ Open.

[R8] Coker E, Kizito S (2018). A narrative review on the human health effects of ambient air pollution in Sub-Saharan Africa: an urgent need for health effects studies. Int J Environ Res Public Health.

[R9] Fisher S, Bellinger DC, Cropper ML, Kumar P, Binagwaho A, Koudenoukpo JB, Park Y, Taghian G, Landrigan PJ (2021). Air pollution and development in Africa: impacts on health, the economy, and human capital. Lancet Planet Health.

[R10] Ganin Y, Lempitsky V (2015). Unsupervised domain adaptation by backpropagation.

[R11] Gunning D, Stefik M, Choi J, Miller T, Stumpf S, Yang G-Z (2019). Xai—explainable artificial intelligence. Sci Robot.

[R12] Handschuh J, Erbertseder T, Schaap M, Baier F (2022). Estimating pm2. 5 surface concentrations from aod: a combination of slstr and modis. Remote Sens Appl Soc Environ.

[R13] He K, Zhang X, Ren S, Sun J (2015). Deep residual learning for image recognition. CoRR.

[R14] He K, Zhang X, Ren S, Sun J (2016). Deep residual learning for image recognition.

[R15] Jiménez-Luna J, Grisoni F, Schneider G (2020). Drug discovery with explainable arti-ficial intelligence. Nat Mach Intell.

[R16] Jin L, Berman JD, Warren JL, Levy JI, Thurston G, Zhang Y, Xu X, Wang S, Zhang Y, Bell ML (2019). A land use regression model of nitrogen dioxide and fine particulate matter in a complex urban core in Lanzhou, China. Environ Res.

[R17] Johnson M, Isakov V, Touma J, Mukerjee S, Özkaynak H (2010). Evaluation of land-use regression models used to predict air quality concentrations in an urban area. Atmos Environ.

[R18] Kang Y, Choi H, Im J, Park S, Shin M, Song C-K, Kim S (2021). Estimation of surface-level no2 and o3 concentrations using tropomi data and machine learning over East Asia. Environ Pollut.

[R19] Lee M, Brauer M, Wong P, Tang R, Tsui TH, Choi C, Cheng W, Lai P-C, Tian L, Thach T-Q (2017). Land use regression modelling of air pollution in high density high rise cities: a case study in Hong Kong. Sci Total Environ.

[R20] Li L, Tang S, Zhang Y, Deng L, Tian Q (2017). Gla: global–local attention for image description. IEEE Trans Multimed.

[R21] Martin RV, Brauer M, van Donkelaar A, Shaddick G, Narain U, Dey S (2019). No one knows which city has the highest concentration of fine particulate matter. Atmos Environ.

[R22] Neyshabur B, Bhojanapalli S, Mcallester D, Srebro N, Guyon I, Luxburg UV, Bengio S, Wallach H, Fergus R, Vishwanathan S, Garnett R (2017). Advances in Neural Information Processing Systems.

[R23] OECD (2016). The economic consequences of outdoor air pollution.

[R24] OpenStreetMap contributors (2017). https://planet.osm.org.

[R25] Oshri B, Hu A, Adelson P, Chen X, Dupas P, Weinstein J, Burke M, Lobell D, Ermon S (2018). Infrastructure quality assessment in Africa using satellite imagery and deep learning.

[R26] Pan SJ, Yang Q (2010). A survey on transfer learning. IEEE Trans Knowl Data Eng.

[R27] Rolf E, Proctor J, Carleton T, Bolliger I, Shankar V, Ishihara M, Recht B, Hsiang S (2021). A generalizable and accessible approach to machine learning with global satellite imagery. Nat Commun.

[R28] Roy R (2016). The cost of air pollution in Africa OECD Development Centre Working Papers, No. 333.

[R29] Selvaraju RR, Cogswell M, Das A, Vedantam R, Parikh D, Batra D (2017). Gradcam: visual explanations from deep networks via gradient-based localization.

[R30] Shorten C, Khoshgoftaar TM (2019). A survey on image data augmentation for deep learning. J Big Data.

[R31] Tan C, Sun F, Kong T, Zhang W, Yang C, Liu C (2018). A survey on deep transfer learning.

[R32] Tzeng E, Hoffman J, Saenko K, Darrell T (2017). Adversarial discriminative domain adaptation.

[R33] UNICEF (2019). S. suffocation in Africa: Air pollution is agrowing menace.

[R34] Wilson AG, Izmailov P (2020). Bayesian deep learning and a probabilistic perspective of generalization. Adv Neural Inf Process Syst.

[R35] Wilson G, Cook DJ (2020). A survey of unsupervised deep domain adaptation. ACM Trans Intell Syst Technol.

[R36] Yadav N (2022). Machine learning for Earth system science and engineering - critical challenges - ProQuest — proquest.com.

[R37] Yeh C, Perez A, Driscoll A, Azzari G, Tang Z, Lobell D, Ermon S, Burke M (2020). Using publicly available satellite imagery and deep learning to understand economic well-being in Africa. Nat Commun.

[R38] Yuan Q, Shen H, Li T, Li Z, Li S, Jiang Y, Xu H, Tan W, Yang Q, Wang J (2020). Deep learning in environmental remote sensing: achievements and challenges. Remote Sens Environ.

[R39] Zhu XX, Tuia D, Mou L, Xia G-S, Zhang L, Xu F, Fraundorfer F (2017). Deep learning in remote sensing: a comprehensive review and list of resources. IEEE Geosci Remote Sens Mag.

[R40] Zhuang F, Qi Z, Duan K, Xi D, Zhu Y, Zhu H, Xiong H, He Q (2020). A compre-hensive survey on transfer learning. Proc IEEE.

