# Supplementary material for: Using deep transfer learning and satellite imagery to estimate urban air quality in data-poor regions
Source: Environ Pollut. Author manuscript; Available in PMC 2024 Feb 1. (PMC7615387; doi:10.1016/j.envpol.2023.122914)
Supplement: Supplementary information [file EMS192497-supplement-Supplementary_information.pdf]

Supplementary Information

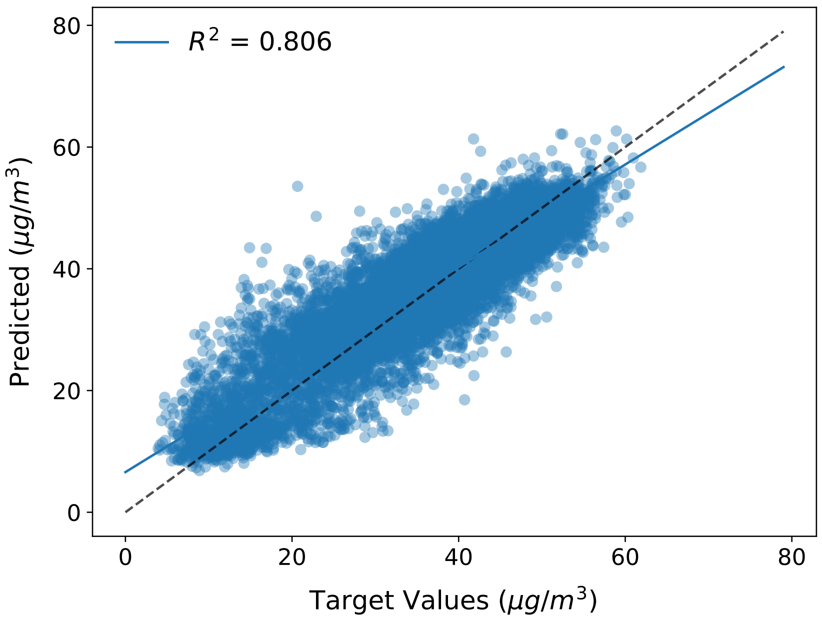

Fig. S1. Predicted (by ResNet-34) vs. Target mean annual NO_2 ($\mu g/m^3$) distribution over the Los Angeles Area (LA).

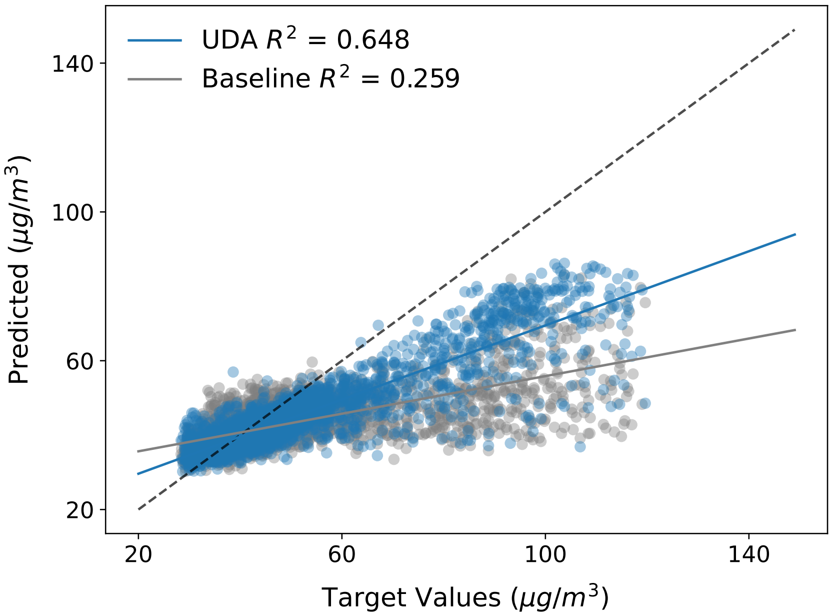

Fig. S2. DeepAQ Predicted vs Target mean annual NO_2 ($\mu g/m^3$) distribution over New York City.

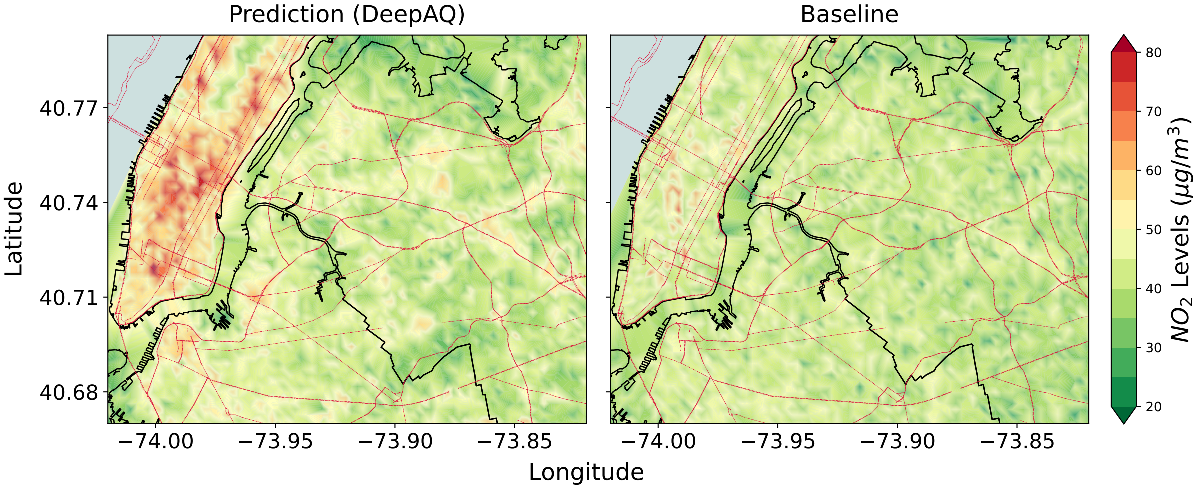

Fig. S3. Mean annual NO_2 levels ($\mu g/m^3$) over New York City (NYC) as predicted by DeepAQ, and compared with the baseline ResNet-34 model (no domain adaptation). In this case, no road network information is provided to the model. The DeepAQ model is able to better identify regions of high NO_2 levels, such as in the Manhattan area of NYC (upper left); however, the performance is poorer compared to the case where road network information is provided explicitly. A possible explanation could be that NO_2 levels are highly correlated with proximity to roads in a large city like NYC, where vehicular emission might be the predominant source of NO_2.

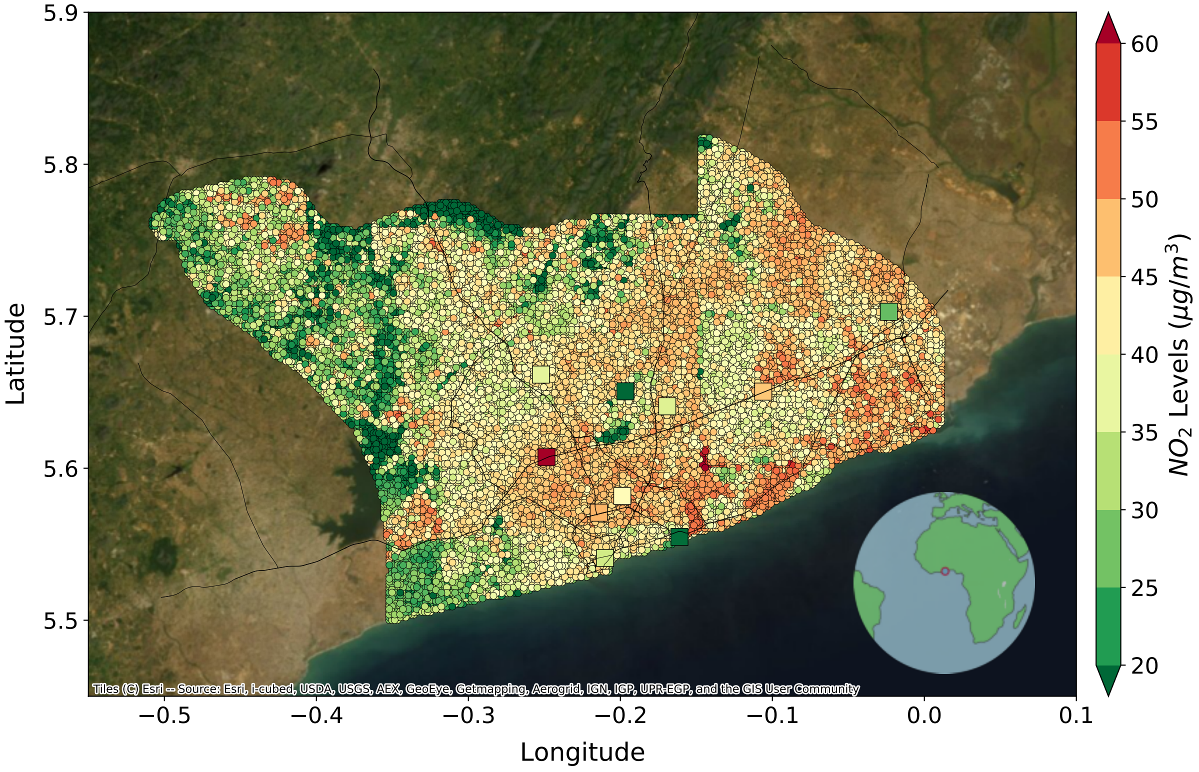

Fig. S4. Mean annual NO_2 levels ($\mu g/m^3$) over Accra, Ghana, as predicted by DeepAQ. In this case, no road network information is provided to the model. The DeepAQ model is still able to capture the spatial distribution of NO_2 levels compared to the case where road network information is explicitly provided to the model.

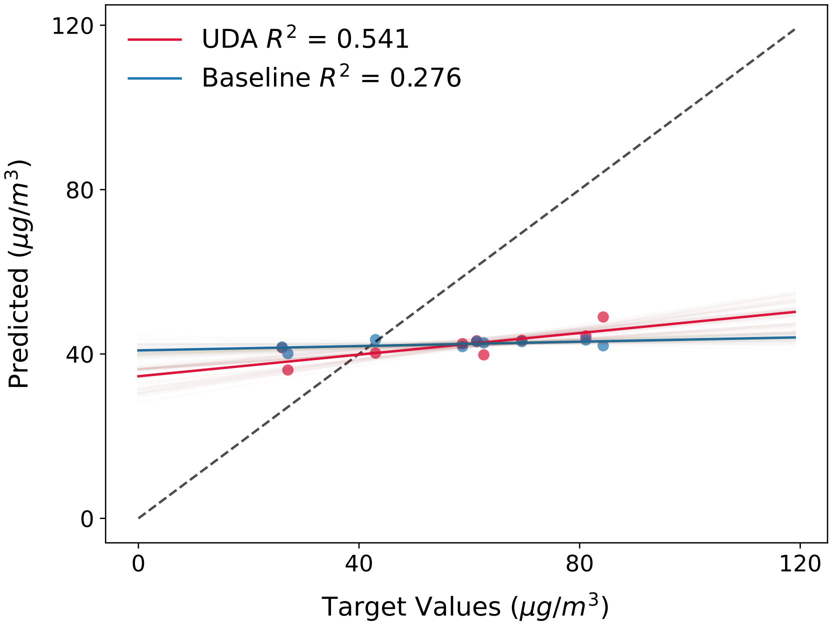

Fig. S5. DeepAQ Predicted vs Target mean annual NO_2 ($\mu g/m^3$) distribution over Accra, Ghana.

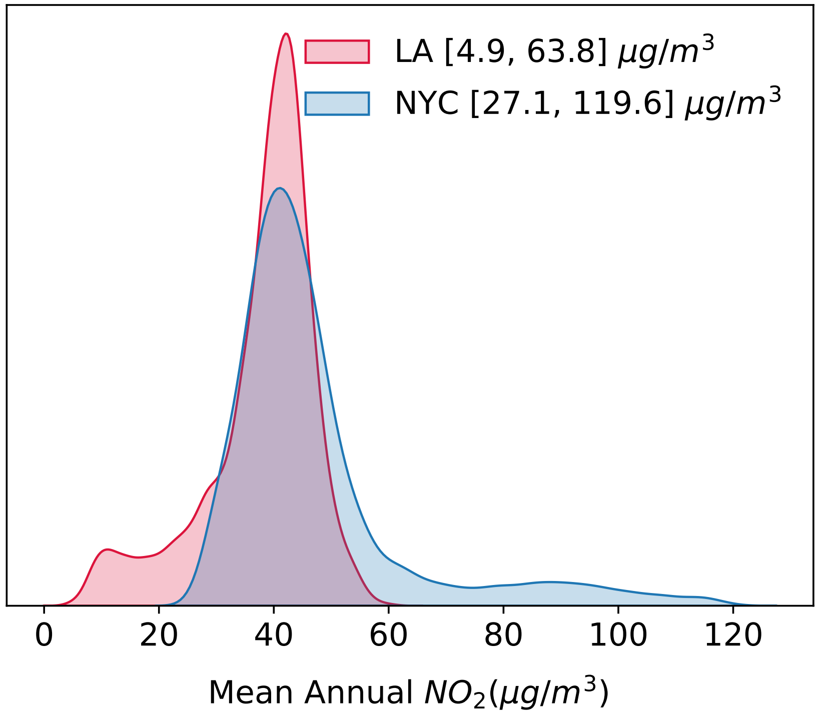

Fig. S6. The difference in the distribution of mean annual NO_2 ($\mu g/m^3$) between LA (source) and NYC (target).
